# Poly[(μ_2_-2-hydr­oxy-2-methyl­propionato-κ^3^ *O* ^1^,*O* ^2^:*O* ^1′^)(μ_2_-2-hydr­oxy-2-methyl­propionato-κ^2^ *O* ^1^:κ*O* ^1′^)dioxido­uranium(VI)]

**DOI:** 10.1107/S1600536809006059

**Published:** 2009-03-06

**Authors:** Takashi Yoshimura, Hidetoshi Kikunaga, Atsushi Shinohara

**Affiliations:** a Department of Chemistry, Graduate School of Science, Osaka University, Toyonaka, Osaka 560-0043, Japan

## Abstract

In the title compound, [UO_2_(C_4_H_7_O_3_)_2_]_*n*_, the dioxouranium(VI) units are linked by 2-hydr­oxy-2-methyl­propionate ligands into a honeycomb structure. The U atom is seven-coordinate in a penta­gonal-bipyramidal geometry. The uncoordinated hydr­oxy groups of the 2-hydr­oxy-2-methyl­propionate ions inter­act with the O atom of the uranyl and with the coordinated hydr­oxy group of an adjacent 2-hydr­oxy-2-methyl­propionate ion through O—H⋯O hydrogen bonds.

## Related literature

For related structures, see: Back *et al.* (2007 ▶); Bombieri *et al.* (1973 ▶, 1974 ▶); Jiang *et al.* (2002 ▶); Thuéry (2006 ▶, 2007*a* ▶,*b* ▶,*c* ▶, 2008 ▶); Xie *et al.* (2003 ▶); Yokoyama *et al.* (1990 ▶).
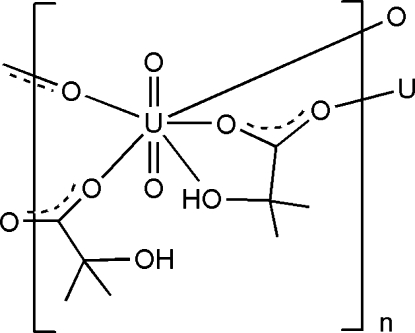


## Experimental

### 

#### Crystal data

[U(C_4_H_7_O_3_)_2_O_2_]
*M* *_r_* = 476.22
Monoclinic, 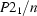

*a* = 9.009 (2) Å
*b* = 8.237 (2) Å
*c* = 17.552 (6) Å
β = 98.246 (9)°
*V* = 1289.0 (6) Å^3^
*Z* = 4
Mo *K*α radiation
μ = 12.62 mm^−1^
*T* = 200 K
0.20 × 0.11 × 0.03 mm

#### Data collection

Rigaku R-AXIS RAPID Imaging Plate diffractometer
Absorption correction: multi-scan (*ABSCOR*; Higashi, 1995 ▶) *T*_min_ = 0.233, *T*_max_ = 0.685
11887 measured reflections
2949 independent reflections
2547 reflections with *I* > 2σ(*I*)
*R*_int_ = 0.050

#### Refinement

*R*[*F* ^2^ > 2σ(*F* ^2^)] = 0.029
*wR*(*F* ^2^) = 0.100
*S* = 0.86
2949 reflections
160 parameters
H-atom parameters constrained
Δρ_max_ = 0.99 e Å^−3^
Δρ_min_ = −2.15 e Å^−3^

### 

Data collection: *PROCESS-AUTO* (Rigaku, 1998 ▶); cell refinement: *PROCESS-AUTO*; data reduction: *TEXSAN* (Rigaku/MSC, 2004 ▶); program(s) used to solve structure: *SIR92* (Altomare *et al.*, 1994 ▶); program(s) used to refine structure: *SHELXL97* (Sheldrick, 2008 ▶); molecular graphics: *ORTEP-3* (Farrugia, 1997 ▶) and *Mercury* (Macrae *et al.*, 2006 ▶); software used to prepare material for publication: *TEXSAN*.

## Supplementary Material

Crystal structure: contains datablocks global, I. DOI: 10.1107/S1600536809006059/ng2546sup1.cif

Structure factors: contains datablocks I. DOI: 10.1107/S1600536809006059/ng2546Isup2.hkl

Additional supplementary materials:  crystallographic information; 3D view; checkCIF report

## Figures and Tables

**Table 1:** Selected bond lengths (Å)

U1—O1	1.783 (5)
U1—O2	1.762 (6)
U1—O3	2.444 (5)
U1—O4	2.407 (5)
U1—O5^i^	2.355 (4)
U1—O7	2.346 (5)
U1—O7	2.336 (5)

Symmetry code: (i) 
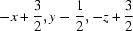
.

**Table 2:** Hydrogen-bond geometry (Å, °)

*D*—H⋯*A*	*D*—H	H⋯*A*	*D*⋯*A*	*D*—H⋯*A*
O3—H13⋯O6	0.82	1.93	2.597 (6)	138
O6—H14⋯O1^ii^	0.82	2.00	2.777 (6)	158

Symmetry code: (ii) 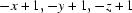.

**Fig. 1.**
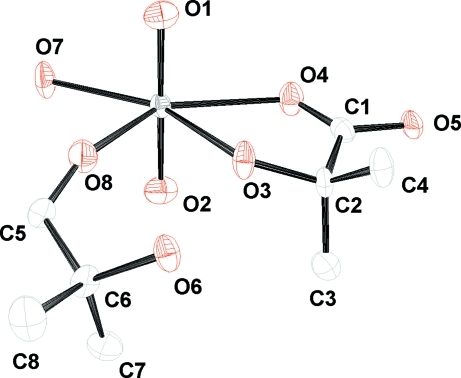
The assymmetric unit of I, with the atom-numbering scheme and displacement ellipsoids drawn at 50% probability level. Hydrogen atoms are omitted for clarity.

**Fig. 2.**
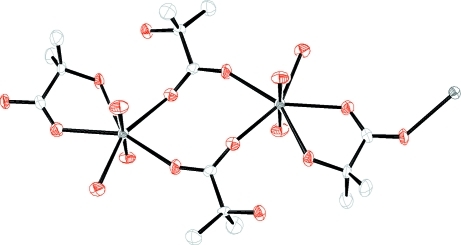
Fragment of the polymeric structure. Hydrogen atoms are omitted for clarity.

**Fig. 3.**
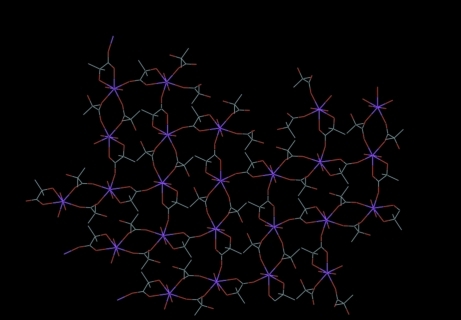
View of the polymeric structure. Hydrogen atoms are omitted for clarity.

## supplementary crystallographic information

### Comment

Structural chemistry of uranyl(VI) complexes with hydroxycarboxylate or alkoxycarboxylate has been extensively studied (Back *et al.* (2007); Bombieri *et al.*(1973, 1974); Jiang *et al.* (2002); Thuéry (2006, 2007*a*,*b*,*c*, 2008); Xie *et al.* (2003); Yokoyama *et al.*(1990)). The crystals of the title compound (I) suitable for single-crystal X-ray analysis were obtained by the reaction of bis(acetato)dioxouranium dihydrate with an excess amount of 2-hydroxy-2-methylpropionic acid in water. Herein, we report on the crystal structure of I. Uranium(VI) atom is seven-coordinate in a pentagonal-bipyramidal structure. The two oxygen atoms are located at the axial positions with nearly linear O(1)—U(1)—O(2) angle (178.3 (2)°). The equatorial positions are coordinated by five oxygen atoms of 2-hydroxy-2-methylpropionate (HIB) ligands. Two kinds of HIB ligands exist in the asymmetric unit. One of the HIB ligands links two uranium atoms by the carboxyl group. The other chelates one uranium atom through the hydroxy and carboxyl groups, moreover the carboxyl group bridges the neighboring uranium atom. As a result, a two-dimensional honeycomb structure is formed. The IR spectrum of I shows stretching bands of the carboxyl group of HIB at 1614 and 1561 cm^-1^.

### Experimental

2-Hydroxy-2-methylpropionic acid (150 mg, 1.45 mmol) was added to a solution of bis(acetato)dioxouranium dihydrate (50 mg, 0.12 mmol) in 3 ml of water. The resulting yellow solution was left for several days at room temperature to give yellow crystals, which were filtered off, washed with a small amount of water, and then dried in air.

### Refinement

H atoms bonded to C and O atoms were placed at calculated positions [C—H = 0.96 and O—H = 0.82] and refined as riding with *U*_iso_(H) = 1.0 *U*_eq_(C,*O*). The deepest hole is 0.68 Å from atom U(1).

### Crystal data

**Table:**

[U(C_4_H_7_O_3_)_2_O_2_]	*F*(000) = 872.00
*M**_r_* = 476.22	*D*_x_ = 2.454 Mg m^−^^3^
Monoclinic, *P*2_1_/*n*	Mo *K*α radiation, λ = 0.7107 Å
Hall symbol: -P 2yn	Cell parameters from 8716 reflections
*a* = 9.009 (2) Å	θ = 3.0–27.5°
*b* = 8.237 (2) Å	µ = 12.62 mm^−^^1^
*c* = 17.552 (6) Å	*T* = 200 K
β = 98.246 (9)°	Platelet, yellow
*V* = 1289.0 (6) Å^3^	0.20 × 0.11 × 0.03 mm
*Z* = 4	

### Data collection

**Table:**

Rigaku R-AXIS RAPID Imaging Plate diffractometer	2949 independent reflections
Radiation source: fine-focus sealed tube	2547 reflections with *I* > 2σ(*I*)
graphite	*R*_int_ = 0.050
Detector resolution: 10.00 pixels mm^-1^	θ_max_ = 27.5°, θ_min_ = 3.0°
ω scans	*h* = 12→11
Absorption correction: multi-scan (*ABSCOR*; Higashi, 1995)	*k* = −9→10
*T*_min_ = 0.233, *T*_max_ = 0.685	*l* = −22→22
11887 measured reflections	

### Refinement

**Table:**

Refinement on *F*^2^	Secondary atom site location: difference Fourier map
Least-squares matrix: full	Hydrogen site location: inferred from neighbouring sites
*R*[*F*^2^ > 2σ(*F*^2^)] = 0.029	H-atom parameters constrained
*wR*(*F*^2^) = 0.100	*w* = 1/[σ^2^(*F*_o_^2^) + (0.079*P*)^2^ + 5.354*P*] where *P* = (*F*_o_^2^ + 2*F*_c_^2^)/3
*S* = 0.86	(Δ/σ)_max_ = 0.001
2949 reflections	Δρ_max_ = 0.99 e Å^−^^3^
160 parameters	Δρ_min_ = −2.15 e Å^−^^3^
Primary atom site location: structure-invariant direct methods	

### Special details

**Table:**

Geometry. All e.s.d's (except the e.s.d. in the dihedral angle between two l.s. planes) are estimated using the full covariance matrix. The cell e.s.d.'s are taken into account individually in the estimation of e.s.d.'s in distances, angles and torsion angles; correlations between e.s.d.'s in cell parameters are only used when they are defined by crystal symmetry. An approximate (isotropic) treatment of cell e.s.d.'s is used for estimating e.s.d.'s involving l.s. planes.
Refinement. Refinement of *F*^2^ against ALL reflections. The weighted *R*-factor *wR* and goodness of fit *S* are based on *F*^2^, conventional *R*-factors *R* are based on *F*, with *F* set to zero for negative *F*^2^. The threshold expression of *F*^2^ > σ(*F*^2^) is used only for calculating *R*-factors(gt) *etc*. and is not relevant to the choice of reflections for refinement. *R*-factors based on *F*^2^ are statistically about twice as large as those based on *F*, and *R*- factors based on ALL data will be even larger.

### Fractional atomic coordinates and isotropic or equivalent isotropic displacement parameters (Å^2^)

**Table:**

	*x*	*y*	*z*	*U*_iso_*/*U*_eq_	
U(1)	0.63772 (2)	0.19668 (3)	0.62425 (1)	0.0180 (1)	
O(1)	0.7538 (6)	0.3101 (5)	0.5695 (3)	0.028 (1)	
O(2)	0.5188 (6)	0.0891 (6)	0.6778 (3)	0.032 (1)	
O(3)	0.4753 (5)	0.4342 (5)	0.6250 (3)	0.026 (1)	
O(4)	0.6920 (5)	0.3869 (6)	0.7286 (3)	0.029 (1)	
O(5)	0.6435 (5)	0.6018 (6)	0.7991 (3)	0.0252 (10)	
O(6)	0.2338 (5)	0.4208 (5)	0.5248 (3)	0.0241 (10)	
O(7)	0.7009 (6)	−0.0415 (6)	0.5630 (3)	0.034 (1)	
O(8)	0.4514 (6)	0.1975 (5)	0.5169 (3)	0.030 (1)	
C(1)	0.6115 (7)	0.5015 (8)	0.7435 (3)	0.022 (1)	
C(2)	0.4611 (7)	0.5295 (8)	0.6929 (4)	0.021 (1)	
C(3)	0.3351 (8)	0.463 (1)	0.7324 (4)	0.036 (2)	
C(4)	0.4392 (10)	0.7064 (7)	0.6704 (5)	0.030 (2)	
C(5)	0.3241 (7)	0.1601 (8)	0.4820 (4)	0.020 (1)	
C(6)	0.1889 (7)	0.2626 (8)	0.4949 (4)	0.021 (1)	
C(7)	0.1191 (10)	0.1790 (9)	0.5588 (5)	0.039 (2)	
C(8)	0.0781 (10)	0.280 (1)	0.4211 (5)	0.038 (2)	
H(1)	0.3277	0.5253	0.7779	0.0356*	
H(2)	0.2423	0.4698	0.6980	0.0356*	
H(3)	0.3555	0.3516	0.7462	0.0356*	
H(4)	0.4388	0.7711	0.7159	0.0297*	
H(5)	0.3454	0.7192	0.6374	0.0297*	
H(6)	0.5196	0.7409	0.6438	0.0297*	
H(7)	0.0321	0.2384	0.5684	0.0386*	
H(8)	0.1908	0.1757	0.6048	0.0386*	
H(9)	0.0907	0.0703	0.5432	0.0386*	
H(10)	−0.0100	0.3359	0.4322	0.0383*	
H(11)	0.0507	0.1746	0.4007	0.0383*	
H(12)	0.1237	0.3413	0.3841	0.0383*	
H(13)	0.4210	0.4724	0.5878	0.0255*	
H(14)	0.2178	0.4883	0.4902	0.0241*	

### Atomic displacement parameters (Å^2^)

**Table:**

	*U*^11^	*U*^22^	*U*^33^	*U*^12^	*U*^13^	*U*^23^
U(1)	0.0192 (2)	0.0158 (2)	0.0185 (2)	0.00083 (8)	0.0005 (1)	−0.00114 (8)
O(1)	0.022 (3)	0.030 (3)	0.032 (3)	0.007 (2)	0.004 (2)	0.007 (2)
O(2)	0.029 (3)	0.025 (2)	0.044 (3)	0.005 (2)	0.009 (2)	0.007 (2)
O(3)	0.034 (3)	0.022 (2)	0.017 (2)	0.008 (2)	−0.008 (2)	−0.005 (2)
O(4)	0.029 (3)	0.025 (2)	0.030 (2)	0.007 (2)	−0.004 (2)	−0.006 (2)
O(5)	0.028 (2)	0.027 (2)	0.020 (2)	−0.008 (2)	0.001 (2)	−0.010 (2)
O(6)	0.030 (2)	0.016 (2)	0.023 (2)	0.002 (2)	−0.005 (2)	−0.001 (2)
O(7)	0.032 (3)	0.027 (3)	0.038 (3)	0.009 (2)	−0.008 (2)	−0.018 (2)
O(8)	0.023 (3)	0.033 (3)	0.033 (3)	0.005 (2)	−0.002 (2)	−0.008 (2)
C(1)	0.023 (3)	0.024 (3)	0.017 (3)	−0.007 (3)	0.000 (2)	−0.001 (3)
C(2)	0.027 (3)	0.016 (3)	0.020 (3)	0.000 (3)	0.000 (2)	−0.008 (2)
C(3)	0.032 (4)	0.042 (4)	0.035 (4)	−0.012 (3)	0.009 (3)	−0.013 (3)
C(4)	0.040 (4)	0.015 (3)	0.032 (4)	0.004 (3)	−0.001 (3)	−0.004 (3)
C(5)	0.022 (3)	0.019 (3)	0.020 (3)	0.009 (3)	0.001 (2)	0.002 (3)
C(6)	0.021 (3)	0.018 (3)	0.022 (3)	0.013 (3)	−0.003 (2)	0.003 (3)
C(7)	0.035 (4)	0.036 (4)	0.048 (5)	−0.015 (3)	0.017 (4)	−0.005 (3)
C(8)	0.035 (4)	0.035 (4)	0.040 (4)	0.014 (3)	−0.012 (4)	−0.004 (4)

### Geometric parameters (Å, °)

**Table:**

U(1)—O(1)	1.783 (5)	C(2)—C(4)	1.514 (9)
U(1)—O(2)	1.762 (6)	C(3)—H(1)	0.960
U(1)—O(3)	2.444 (5)	C(3)—H(2)	0.960
U(1)—O(4)	2.407 (5)	C(3)—H(3)	0.960
U(1)—O(5)^i^	2.355 (4)	C(4)—H(4)	0.960
U(1)—O(7)	2.346 (5)	C(4)—H(5)	0.960
U(1)—O(8)	2.336 (5)	C(4)—H(6)	0.960
O(3)—C(2)	1.449 (8)	C(5)—C(6)	1.525 (10)
O(3)—H(13)	0.820	C(6)—C(7)	1.53 (1)
O(4)—C(1)	1.241 (8)	C(6)—C(8)	1.523 (10)
O(5)—C(1)	1.280 (8)	C(7)—H(7)	0.960
O(6)—C(6)	1.441 (8)	C(7)—H(8)	0.960
O(6)—H(14)	0.820	C(7)—H(9)	0.960
O(7)—C(5)^ii^	1.257 (8)	C(8)—H(10)	0.960
O(8)—C(5)	1.259 (8)	C(8)—H(11)	0.960
C(1)—C(2)	1.528 (8)	C(8)—H(12)	0.960
C(2)—C(3)	1.51 (1)		
			
O(1)—U(1)—O(2)	178.3 (2)	C(1)—C(2)—C(4)	111.6 (6)
O(1)—U(1)—O(3)	88.9 (2)	C(3)—C(2)—C(4)	112.9 (6)
O(1)—U(1)—O(4)	89.9 (2)	C(2)—C(3)—H(1)	109.5
O(1)—U(1)—O(5)^i^	88.5 (2)	C(2)—C(3)—H(2)	109.5
O(1)—U(1)—O(7)	89.5 (2)	C(2)—C(3)—H(3)	109.5
O(1)—U(1)—O(8)	88.5 (2)	H(1)—C(3)—H(2)	109.5
O(2)—U(1)—O(3)	89.4 (2)	H(1)—C(3)—H(3)	109.5
O(2)—U(1)—O(4)	89.7 (2)	H(2)—C(3)—H(3)	109.5
O(2)—U(1)—O(5)^i^	93.0 (2)	C(2)—C(4)—H(4)	109.5
O(2)—U(1)—O(7)	91.7 (2)	C(2)—C(4)—H(5)	109.5
O(2)—U(1)—O(8)	90.6 (2)	C(2)—C(4)—H(6)	109.5
O(3)—U(1)—O(4)	62.1 (1)	H(4)—C(4)—H(5)	109.5
O(3)—U(1)—O(5)^i^	135.7 (2)	H(4)—C(4)—H(6)	109.5
O(3)—U(1)—O(7)	148.9 (2)	H(5)—C(4)—H(6)	109.5
O(3)—U(1)—O(8)	68.9 (2)	O(7)^ii^—C(5)—O(8)	124.4 (6)
O(4)—U(1)—O(5)^i^	73.7 (2)	O(7)^ii^—C(5)—C(6)	116.7 (6)
O(4)—U(1)—O(7)	149.0 (2)	O(8)—C(5)—C(6)	119.0 (6)
O(4)—U(1)—O(8)	131.0 (2)	O(6)—C(6)—C(5)	111.4 (5)
O(5)^i^—U(1)—O(7)	75.3 (2)	O(6)—C(6)—C(7)	105.3 (5)
O(5)^i^—U(1)—O(8)	155.1 (2)	O(6)—C(6)—C(8)	109.8 (6)
O(7)—U(1)—O(8)	80.0 (2)	C(5)—C(6)—C(7)	106.3 (6)
U(1)—O(3)—C(2)	124.2 (3)	C(5)—C(6)—C(8)	111.5 (6)
U(1)—O(3)—H(13)	126.3	C(7)—C(6)—C(8)	112.2 (6)
C(2)—O(3)—H(13)	109.5	C(6)—C(7)—H(7)	109.5
U(1)—O(4)—C(1)	126.5 (4)	C(6)—C(7)—H(8)	109.5
U(1)^iii^—O(5)—C(1)	136.7 (4)	C(6)—C(7)—H(9)	109.5
C(6)—O(6)—H(14)	109.5	H(7)—C(7)—H(8)	109.5
U(1)—O(7)—C(5)^ii^	154.9 (4)	H(7)—C(7)—H(9)	109.5
U(1)—O(8)—C(5)	153.0 (5)	H(8)—C(7)—H(9)	109.5
O(4)—C(1)—O(5)	125.3 (6)	C(6)—C(8)—H(10)	109.5
O(4)—C(1)—C(2)	119.3 (5)	C(6)—C(8)—H(11)	109.5
O(5)—C(1)—C(2)	115.4 (6)	C(6)—C(8)—H(12)	109.5
O(3)—C(2)—C(1)	102.7 (5)	H(10)—C(8)—H(11)	109.5
O(3)—C(2)—C(3)	109.9 (5)	H(10)—C(8)—H(12)	109.5
O(3)—C(2)—C(4)	109.3 (5)	H(11)—C(8)—H(12)	109.5
C(1)—C(2)—C(3)	109.9 (5)		

Symmetry codes: (i) −*x*+3/2, *y*−1/2, −*z*+3/2; (ii) −*x*+1, −*y*, −*z*+1; (iii) −*x*+3/2, *y*+1/2, −*z*+3/2.

### Hydrogen-bond geometry (Å, °)

**Table:**

*D*—H···*A*	*D*—H	H···*A*	*D*···*A*	*D*—H···*A*
O(3)—H(13)···O(6)	0.820	1.927	2.597 (6)	138.188
O(6)—H(14)···O(1)^iv^	0.820	1.999	2.777 (6)	158.201

Symmetry codes: (iv) −*x*+1, −*y*+1, −*z*+1.

## References

[bb1] Altomare, A., Cascarano, G., Giacovazzo, C., Guagliardi, A., Burla, M. C., Polidori, G. & Camalli, M. (1994). *J. Appl. Cryst.* **27**, 435.

[bb2] Back, D. F., Manzoni de Oliveira, G. & Schulz Lang, E. (2007). *Z. Anorg. Allg. Chem.* **633**, 729–733.

[bb3] Bombieri, G., Croatto, U., Graziani, R., Forsellini, E. & Magon, L. (1974). *Acta Cryst.* B**30**, 407–411.

[bb4] Bombieri, G., Graziani, R. & Forsellini, E. (1973). *Inorg. Nucl. Chem. Lett.* **9**, 551–557.

[bb5] Farrugia, L. J. (1997). *J. Appl. Cryst.* **30**, 565.

[bb6] Higashi, T. (1995). *ABSCOR* Rigaku Corporation, Tokyo, Japan.

[bb7] Jiang, J., Sarsfield, M. J., Renshaw, J. C., Livens, F. R., Collison, D., Charnock, J. M., Helliwell, M. & Eccles, H. (2002). *Inorg. Chem.* **41**, 2799–2806. 10.1021/ic020121v 12005506

[bb8] Macrae, C. F., Edgington, P. R., McCabe, P., Pidcock, E., Shields, G. P., Taylor, R., Towler, M. & van de Streek, J. (2006). *J. Appl. Cryst.* **39**, 453–457.

[bb9] Rigaku (1998). *PROCESS-AUTO* Rigaku Corporation, Tokyo, Japan.

[bb10] Rigaku/MSC (2004). *TEXSAN* Rigaku/MSC, The Woodlands, Texas, USA.

[bb11] Sheldrick, G. M. (2008). *Acta Cryst.* A**64**, 112–122. 10.1107/S0108767307043930 18156677

[bb12] Thuéry, P. (2006). *Chem. Commun.* pp. 853–855. 10.1039/b516191f 16479289

[bb13] Thuéry, P. (2007*a*). *CrystEngComm*, **9**, 358–360.

[bb14] Thuéry, P. (2007*b*). *Inorg. Chem.* **46**, 2307–2315. 10.1021/ic061772k 17295470

[bb15] Thuéry, P. (2007*c*). *Polyhedron*, **26**, 101–106.

[bb16] Thuéry, P. (2008). *CrystEngComm*, **10**, 79–85.

[bb17] Xie, Y.-R., Zhao, H., Wang, X.-S., Qu, Z.-R., Xiong, R.-G., Xue, X., Xue, Z. & You, X.-Z. (2003). *Eur. J. Inorg. Chem.* pp. 3712–3715.

[bb18] Yokoyama, Y., Inaba, A., Hara, H., Yamazaki, T., Tamura, H. & Kushi, Y. (1990). *Chem. Lett.* pp. 671–674.

